# ‘Preconditioning’ with Low Dose Lipopolysaccharide Aggravates the Organ Injury / Dysfunction Caused by Hemorrhagic Shock in Rats

**DOI:** 10.1371/journal.pone.0122096

**Published:** 2015-04-01

**Authors:** Regina Sordi, Fausto Chiazza, Nimesh S. A. Patel, Rachel A. Doyle, Massimo Collino, Christoph Thiemermann

**Affiliations:** 1 The William Harvey Research Institute, Barts and The London School of Medicine & Dentistry, Queen Mary University of London, London, United Kingdom; 2 Capes Foundation, Ministry of Education of Brazil, Brasilia/DF, Brazil; 3 Department of Drug Science and Technology, University of Turin, Turin, Italy; Georgia Regents University, UNITED STATES

## Abstract

**Methods:**

Male rats were ‘pretreated’ with phosphate-buffered saline (PBS; i.p.) or LPS (1 mg/kg; i.p.) 24 h prior to HS. Mean arterial pressure (MAP) was maintained at 30 ± 2 mmHg for 90 min or until 25% of the shed blood had to be re-injected to sustain MAP. This was followed by resuscitation with the remaining shed blood. Four hours after resuscitation, parameters of organ dysfunction and systemic inflammation were assessed.

**Results:**

HS resulted in renal dysfunction, and liver and muscular injury. At a first glance, LPS preconditioning attenuated organ dysfunction. However, we discovered that HS-rats that had been preconditioned with LPS (a) were not able to sustain a MAP at 30 mmHg for more than 50 min and (b) the volume of blood withdrawn in these animals was significantly less than in the PBS-control group. This effect was associated with an enhanced formation of the nitric oxide (NO) derived from inducible NO synthase (iNOS). Thus, a further control group in which all animals were resuscitated after 50 min of hemorrhage was performed. Then, LPS preconditioning aggravated both circulatory failure and organ dysfunction. Most notably, HS-rats pretreated with LPS exhibited a dramatic increase in NF-κB activation and pro-inflammatory cytokines.

**Conclusion:**

In conclusion, LPS preconditioning predisposed animals to an earlier vascular decompensation, which may be mediated by an excess of NO production secondary to induction of iNOS and activation of NF-κB. Moreover, LPS preconditioning increased the formation of pro-inflammatory cytokines, which is likely to have contributed to the observed aggravation of organ injury/dysfunction caused by HS.

## Introduction

Severe hemorrhage can lead to a state of global ischemia and shock ultimately resulting in 40% of all trauma-related deaths. Resuscitation causes an ischemia-reperfusion-type organ injury as well as systemic inflammation, both of which ultimately lead to the development of multiple organ failure, which in turn is a key driver for the high mortality rate in these patients [1].

Hemorrhagic shock (HS) is essentially characterized by 2 phases: (i) a compensatory phase, which is the attempt of the body to maintain a normal blood pressure by releasing endogenous vasoconstrictors such as norepinephrine and angiotensin II and (ii) a decompensatory phase, which occurs after the development of hyporeactivity to the vasoconstrictors and it is characterized by a progressive vasodilatation that ultimately results in death [2]. Nitric oxide (NO) overproduction by inducible NO synthase (iNOS) importantly contributes to vascular hyporeactivity and vascular decompensation following HS [3, 4]. On the other hand, a small amount of NO is pivotal for the preservation of microvascular perfusion [5].

There is now good evidence that pre-treatment or ‘preconditioning’ with low doses of endotoxin (lipopolysaccharide or LPS) reduces the injury/dysfunction caused by a subsequent challenge with ischemia or endotoxemia, the latter often being referred to as ‘endotoxin tolerance’. Endotoxin tolerance was first reported in 1946 as a phenomenon in which the pretreatment of rabbits with small amounts of LPS attenuated the fever and the mortality caused by a subsequent larger LPS challenge [6, 7]. Today we know that the phenomenon of tolerance also applies to stimuli other than endotoxin such as other toll-like receptor ligands, pro-inflammatory cytokines and even ischemia (see [8]). For instance, the exposure of rats to a small dose of LPS attenuates the injury/dysfunction caused by ischemia of the heart, kidney, lung, liver and the brain [9–16].

As a) preconditioning with LPS attenuates the injury and dysfunction of many different organs subjected to ischemia-reperfusion (see above), and b) as HS leads to organ ischemia (and resuscitation to reperfusion injury) resulting in the injury/dysfunction of multiple organs, the aim of the present study was to investigate the effects of LPS-preconditioning in a rat model of severe HS and organ injury/dysfunction. Surprisingly, we have discovered that preconditioning with a single dose of LPS enhanced, rather than reduced, the organ injury and dysfunction associated with HS. We have subsequently investigated possible mechanisms by which preconditioning with LPS aggravated the condition.

## Materials and Methods

### Ethics Statement

The animal protocols used in this study were approved by the Animal Welfare Ethics Review Board (AWERB) of Queen Mary University of London (PPL: 70/7348) in accordance with the derivatives of Home Office guidance on Operation of Animals (Scientific Procedures Act 1986) published by Her Majesty’s Stationery Office and the Guide for the Care and Use of Laboratory Animals of the National Research Council.

### Hemorrhagic shock model

This study was carried out on 45 male Wistar rats (Charles River Ltd, Margate, UK) weighing 230 to 280 g receiving a standard diet and water *ad libitum*. Rats were anesthetized with sodium thiopentone (120 mg/kg i.p. maintained using ~ 10 mg/kg i.v. as required) and the trachea, bladder (to collect urine) and jugular vein (to administer compounds and blood) were cannulated. Body temperature was monitored by a rectal thermometer and maintained at 37 ± 0.5 by means of a homeothermic blanket system (Harvard Apparatus, Natick, MA). Heparinized (50 U/mL) polyethylene catheters were inserted into the left femoral artery for recording mean arterial pressure (MAP) and into the right carotid artery for blood withdrawal. Blood was withdrawn at 1 mL/min to achieve a fall in MAP to 30 ± 2 mmHg, which was recorded with a pressure transducer coupled to a Powerlab 8/30 (AD Instruments Pty Ltd., Castle Hill, Australia). MAP was maintained at 30 ± 2 mmHg either by further withdrawal of blood during the compensation phase or administration of the shed blood during the decompensation phase. At 90 min after initiation of hemorrhage or when 25% of the shed blood had to be re-injected to sustain MAP at 30 ± 2 mmHg, resuscitation was performed with the remaining shed blood mixed with 100 IU/mL heparinized saline plus the same volume of blood spent during decompensation of Ringer’s lactate over a period of 5 min. A group of animals were resuscitated at 50 min after initiation of hemorrhage (HS_50_). One hour after resuscitation an infusion of Ringers Lactate (1.5 mL/kg/h; i.v.) was started as fluid replacement and was maintained throughout the experiment for a total of 4 h. Sham-operated rats were used as control and underwent identical surgical procedures but without hemorrhage or resuscitation.

### Experimental design

Rats were randomly allocated into the following groups: (i) Vehicle + Sham; (ii) LPS + Sham; (iii) Vehicle + HS_90_, (iv) Vehicle + HS_50_ (the group of animals that were resuscitated 50 min after hemorrhage) and (v) LPS + HS. Rats were administered i.p. vehicle (PBS) or LPS (1 mg/kg) 24 h before the HS challenge. The preconditioning protocol used here (e.g. injection of a single dose of LPS of 1 mg/kg i.p. at 24 h prior to a subsequent insult) has been previously shown to protect organs against ischemia-reperfusion injury [9, 11, 15].

### Organ injury assessment

Four hours after the initiation of resuscitation, blood samples were collected via the carotid artery and then were centrifuged to separate serum from which creatinine, aspartate aminotransferase (AST), alanine aminotransferase (ALT) and creatine kinase (CK) were measured within 24 h (IDEXX Laboratories Ltd, West Yorkshire, UK). In addition, during the last 3 h of resuscitation, urine was obtained for the estimation of creatinine clearance.

### Western blotting

Briefly, kidney and liver samples were homogenized in buffer and centrifuged at 4000 rpm for 5 min at 4 ^o^C. To obtain the cytosolic fraction, supernatants were centrifuged at 14000 rpm at 4 ^o^C for 40 min. The pelleted nuclei were re-suspended in extraction buffer and centrifuged at 14000 rpm for 20 min at 4 ^o^C. Protein content was determined on both nuclear and cytosolic extracts using bicinchoninic acid (BCA) protein assay (Thermo Fisher Scientific Inc, Rockford, IL). Proteins were separated by 8% sodium dodecyl sulphatepolyacrylamide gel electrophoresis (SDS-PAGE) and transferred to a polyvinyldenediflouoride (PVDF) membrane, which was incubated with a primary antibody [mouse anti-total IĸBα (1:1000); mouse anti-IκBα pSer32/36 (1:1000); rabbit anti-NF-κB p65 (1:1000); rabbit anti-total iNOS (1:200)]. Membranes were incubated with a secondary antibody conjugated with horseradish peroxidase (1:2000) for 30 min at room temperature and developed with ECL detection system. The immunoreactive bands were visualized by autoradiography and the densitometric analysis was performed using Gel Pro Analyzer 4.5, 2000 software (Media Cybernetics, Silver Spring, MD, USA). The membranes were stripped and incubated with β-actin monoclonal antibody (1:5000) and subsequently with an anti-mouse antibody (1:10000) to assess gel-loading homogeneity. Densitometric analysis of the related bands is expressed as relative optical density, corrected for the corresponding β-actin contents, and normalized using the related sham-operated band.

### Serum nitrite and nitrate (NOx)

Serum nitrite and nitrate (NOx) concentrations were used as an indicator of NO synthesis. Briefly, nitrate was converted to nitrite using nitrate reductase as previously described [17]. Subsequently, the total nitrite in serum was assayed by adding Griess reagent [0.05% (wt/vol) naphthalethylenediamine dihydrochloride and 0.5% (wt/vol) sulphanilamide in 2.5% (vol/vol) phosphoric acid] to each sample and read at 550 nm. Standard curves of nitrite and nitrate were run simultaneously and NOx concentrations were calculated by comparison with standard solution of sodium nitrate prepared in saline. Values are expressed as μM NOx.

### Determination of cytokines in plasma and tissue

Kidney and liver samples were homogenized in phosphate buffer containing 0.5% Triton X-100 and protease inhibitor cocktail (10 μL/mL; P-8340; Sigma-Aldrich Company Ltd, Poole, Dorset, UK). The homogenates were centrifuged at 5000 g at 4 ^o^C for 10 min and the supernatants were aliquoted and stored at -80 ^o^C. The protein content was determined in each sample (after 1:20 dilution in lysis buffer) using BCA protein assay (Thermo Fisher Scientific Inc, Rockford, IL). The pro-inflammatory cytokines TNF-α, IL-6 and IL-1β were determined on the tissue homogenates and serum samples using commercial immunoassay kit (R&D Systems, Minneapolis, MN) according to the manufacturer protocol. The results were expressed as pg/mg of protein (tissue) or pg/mL of serum.

### Reagents

Thiopentone sodium was obtained from Rhone Merieux (Harlow, Essex, UK). LPS (Escherichia coli, serotype 0127:B8) and all other compounds used in this study were purchased from Sigma-Aldrich Company Ltd (Poole, Dorset, UK), unless otherwise stated. All stock solutions were prepared using non-pyrogenic saline (0.9% [wt/vol] NaCl; Baxter Healthcare Ltd, Thetford, Norfolk, UK). Ringer’s lactate was also purchased from Baxter Healthcare. The bicinchoninic acid protein assay kit and SuperBlock blocking buffer were from Thermo Fisher Scientific Inc. (Rockford, IL, USA). Antibodies were from Cell Signalling Technology Inc. (Beverly, MA, USA).

### Statistical analysis

All values are expressed as mean ± SEM. Statistical analysis was carried out using Graph Prism 5.03 (Graph Pad Software, San Diego, CA). Data were assessed by unpaired t-test or two-way ANOVA followed by Bonferroni’s post hoc test. A p-value of less than 0.05 was considered to be significant.

## Results

### Effect of LPS preconditioning on HS

**Fig 1:**
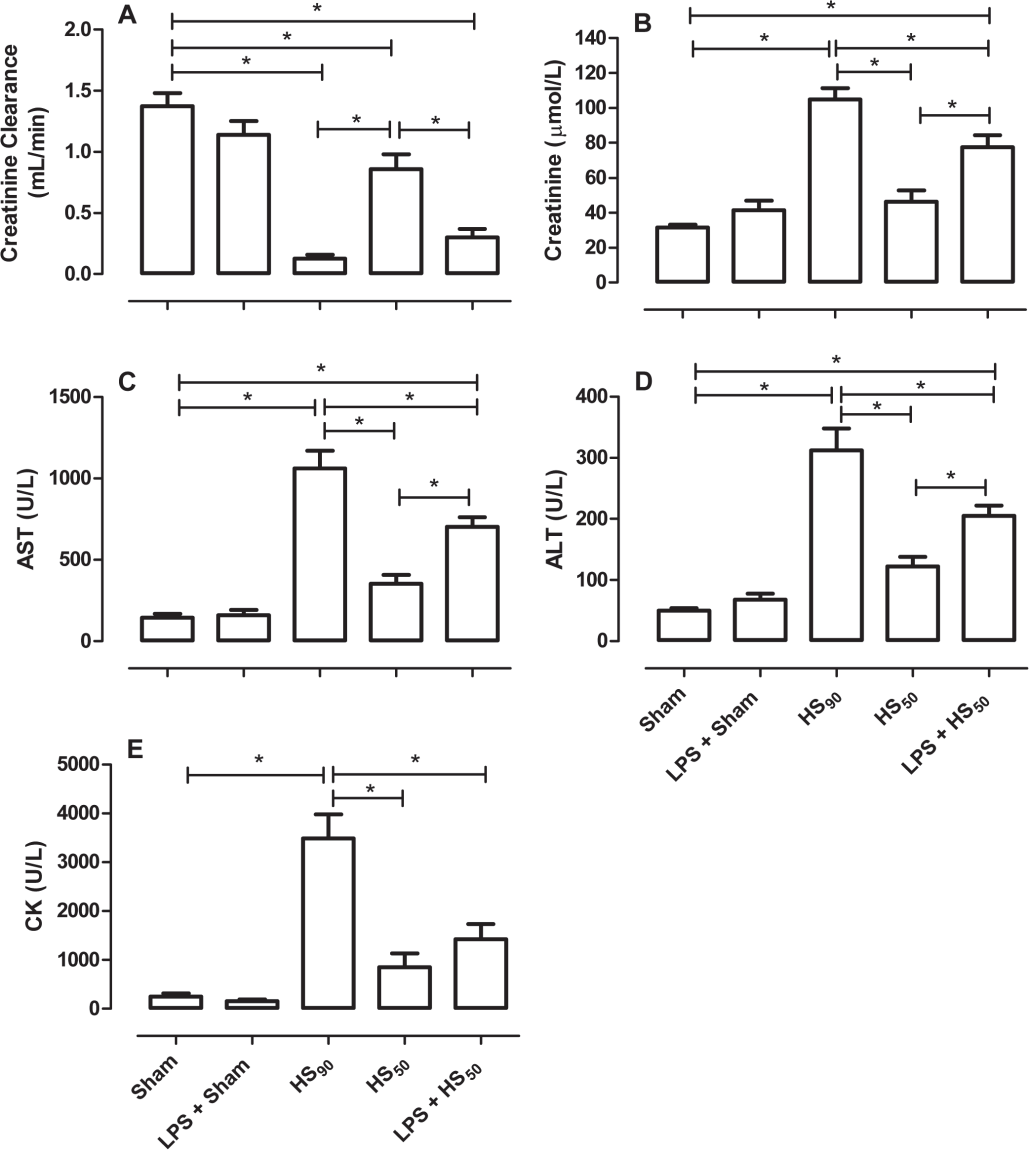
Effect of LPS preconditioning on multiple organ dysfunction induced by HS. Rats were injected vehicle (PBS) or LPS (1 mg/kg) i.p. 24 h prior to HS_90_ or HS_50_. Four hours after resuscitation the (A) creatinine clearance as a measure of glomerular function; (B) serum creatinine level as a measure of renal function; (C) serum aspartate aminotransferase (AST) and (D) serum alanine aminotransferase (ALT) as a measure of liver injury; and (E) serum creatine kinase (CK) level as a measure of neuromuscular injury were determined. Sham animals were used as control. Data are expressed as mean ± SEM of 8–10 animals. Statistical analysis was performed using one-way ANOVA followed by Bonferroni’s post hoc test. * p < 0.05 among the groups.

When compared to sham-operated rats, HS_90_ resulted in a reduction in creatinine clearance (from 1.37 ± 0.11 to 0.12 ± 0.03 mL/min; p<0.05; Fig 1A) and consequently an increased concentration of serum creatinine (from 31.5 ± 1.5 to 104.9 ± 6.3 μmol/L; p<0.05; Fig 1B) and, hence, renal dysfunction. When compared to sham-operated rats, HS_90_ also resulted in significant increases in serum AST (from 143.8 ± 24.6 to 1062 ± 107.5 U/L; p<0.05; Fig 1C) and ALT (from 50.1 ± 4.3 to 312 ± 35.8 U/L; p<0.05; Fig 1D) and, hence, liver injury. HS_90_ also resulted in a significant increase in serum CK (from 254.4 ± 64.2 to 3485 ± 495.6 U/L; p<0.05; Fig 1E), suggesting the development of neuromuscular injury. Preconditioning with LPS attenuated the rises in serum creatinine, CK, AST and ALT (Fig 1B–1E) caused by HS_90_, but had no significant effect on creatinine clearance (Fig 1A).

**Fig 2:**
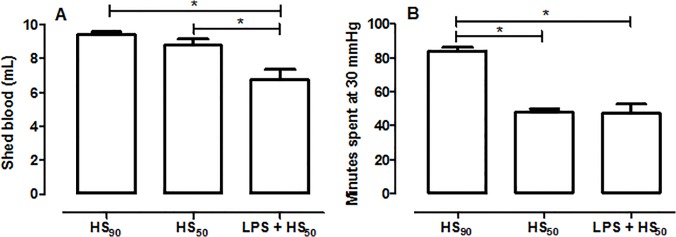
Effect of LPS preconditioning on shed blood and period of time spent at 30 mmHg. Rats were injected vehicle (PBS) or LPS (1 mg/kg) i.p. 24 h prior to HS_90_ or HS_50_. The shed blood (A) and the period of time that animals spent at 30 mmHg (B) are shown. Data are expressed as mean ± SEM of 8–10 animals. Statistical analysis was performed using one-way ANOVA followed by Bonferroni’s post hoc test. * p < 0.05 among the groups.

However, when we evaluated the amount of blood withdrawn to reach a target MAP of 30 ± 2 mmHg and also the length of time that animals spent at this MAP of 30 ± 2 mmHg, we made a surprising discovery. The volume of blood withdrawn during hemorrhage was 9.41 ± 0.18 mL for rats subjected to HS_90_ that had been pretreated with vehicle, and 6.73 ± 0.62 mL for rats subjected to HS that had been pretreated with LPS (p<0.05; Fig 2A). The average rate of resuscitation with shed blood was 1.5 ± 0.07 and 1.0 ± 0.09 mL/min for the HS_90_ and LPS + HS groups, respectively. Moreover, 90% of the HS-animals that had been preconditioned with LPS were unable to maintain an MAP of 30 ± 2 mmHg for 90 min even when 25% of the shed blood had been re-injected (data not shown). Thus, HS-rats pretreated with vehicle spent a longer time at an average MAP of 30 mmHg than HS-rats pretreated with LPS (84 ± 2.1 and 47.2 ± 5.6 minutes, respectively, p<0.05; Fig 2B).

Thus, as animals subjected to LPS-preconditioning were resuscitated 47.2 ± 5.6 min after initiation of hemorrhage (Fig 2B), we performed a further control group in which all animals were resuscitated 50 min after initiation of hemorrhage (HS_50_). The volume of blood withdrawn during hemorrhage in the HS_50_ group was 8.78 ± 0.37 mL (Fig 2A) and the rate of resuscitation with shed blood was 1.6 ± 0.09 mL/min. When compared with sham-operated animals, hemorrhage for 50 min followed by 4 h of resuscitation (HS_50_) caused a small decrease in creatinine clearance (from 1.37 ± 0.11 to 0.86 ± 0.12 mL/min; p<0.05; Fig 1A) but no significantly changes in serum creatinine, AST, ALT or CK were noticed (Fig 1). When compared with animals subjected to 50 min of hemorrhage that had been pretreated with vehicle, animals that had been preconditioned with LPS had a significant reduction in creatinine clearance (from 0.86 ± 0.12 to 0.30 ± 0.07 mL/min; p<0.05; Fig 1A) as well as increased concentrations of serum creatinine (from 46.29 ± 6.5 to 77.6 ± 6.8 μmol/L; p<0.05; Fig 1B), AST (from 351.8 ± 56.3 to 701.7 ± 60.0 U/L; p<0.05; Fig 1C), ALT (from 122.0 ± 16.1 to 205 ± 16.7 U/L; p<0.05; Fig 1D) and CK (from 856.4 ± 279.5 to 1422 ± 311.0 U/L; p<0.05; Fig 1E). Thus, when the length of the period of hemorrhage was identical between animals pretreated with vehicle or LPS, the LPS-preconditioning increased the organ injury/dysfunction caused by HS.

### Effect of LPS preconditioning on circulatory failure

**Fig 3:**
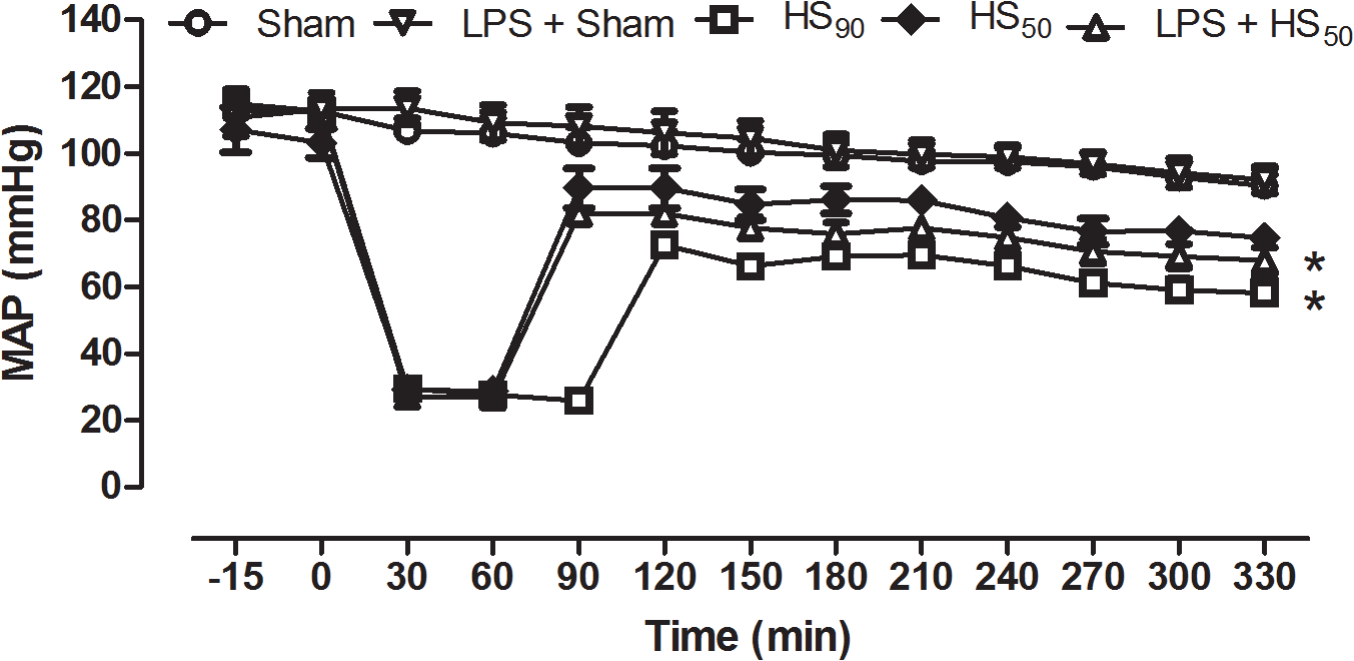
Effect of LPS preconditioning on mean arterial pressure (MAP) of rats. Rats were injected vehicle (PBS) or LPS (1 mg/kg) i.p. 24 h prior to HS_90_ or HS_50_. The MAP was recorded during the whole experiment. Sham animals were used as control. Data are expressed as mean ± SEM of 8–10 animals. Statistical analysis was performed using two-way ANOVA followed by Bonferroni’s post hoc test. *p < 0.05 versus sham group.

The MAP of HS_90_-rats and LPS preconditioned HS_50_-rats, even after resuscitation, remained significantly lower than the MAP values observed in sham-operated rats (p<0.05), suggesting the development of a circulatory failure (late vascular decompensation). In contrast, the MAP of vehicle treated HS_50_-rats was not statistically different from sham group, indicating that LPS preconditioning aggravated the delayed vascular decompensation (Fig 3).

### Effect of LPS preconditioning on iNOS expression and NO production

**Fig 4:**
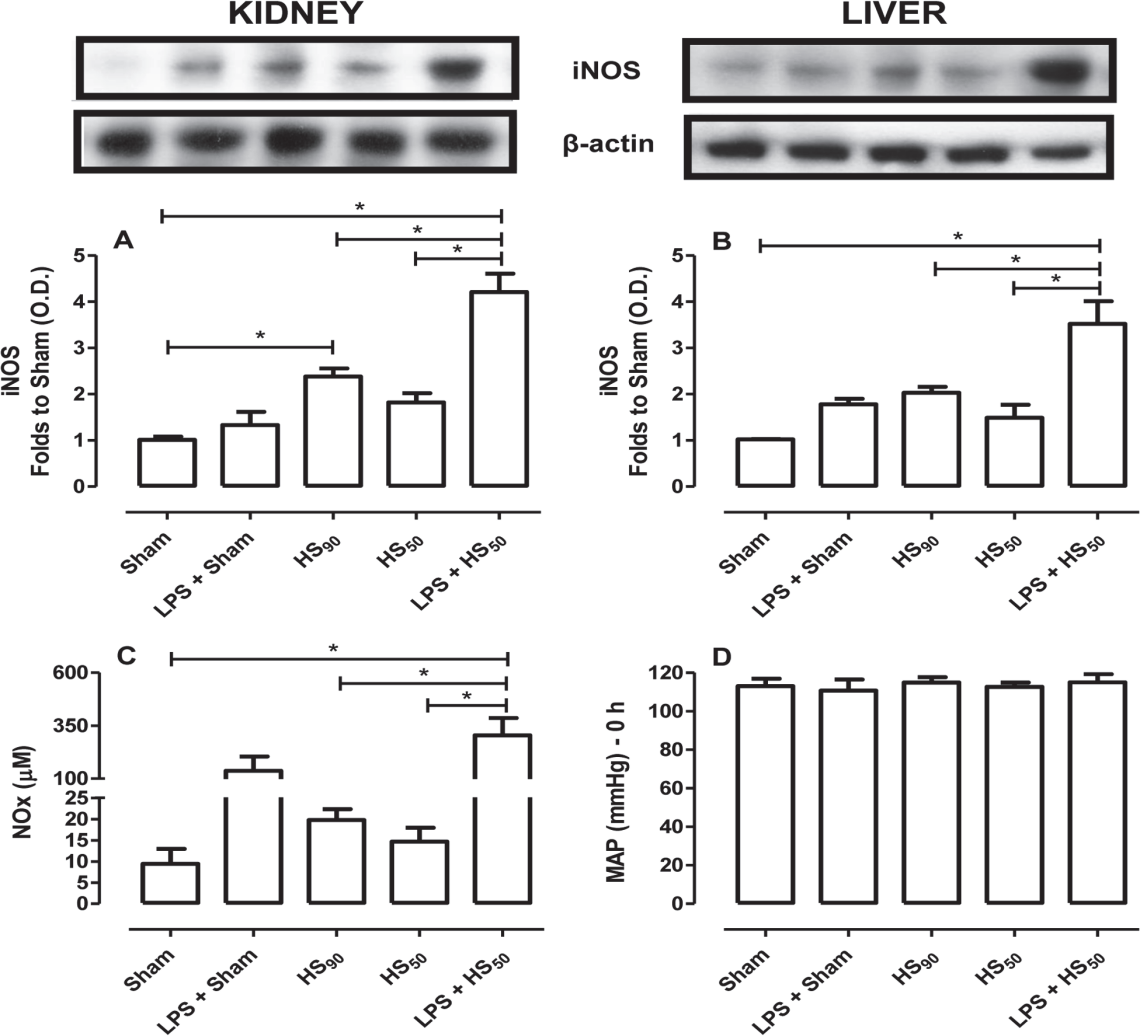
Effect of LPS preconditioning on iNOS expression and serum nitrite and nitrate (NOx). Rats were injected vehicle (PBS) or LPS (1 mg/kg) i.p. 24 h prior to HS_90_ or HS_50_. The iNOS expression on the kidney (A) and liver (B) was determined by western blotting. Protein expression was measured as relative optical density (O.D.), corrected for the corresponding β-actin contents and normalized with the Sham band. (C) The concentration of serum NOx was measured as described at Methods. (D) The basal mean arterial pressure (MAP) of all groups is shown. Data are expressed as mean ± SEM of n observations. Statistical analysis was performed using one-way ANOVA followed by Bonferroni’s post hoc test. *p < 0.05 among the groups.

As an enhanced formation of NO by iNOS is implicated in the decompensation phase of HS [4] and LPS preconditioning animals were not able to sustain their blood pressure at 30 ± 2 mmHg during the 90 min period of hemorrhage, we investigated the role of iNOS and NO production in these animals. As the main organs affected by HS were the kidney and liver, we investigated the expression of iNOS in renal and liver biopsies from all animal groups. When compared with sham-operated rats, kidneys from rats subjected to HS_90_ exhibited a significant increase on iNOS expression (Fig 4A). However, no significant increase in iNOS expression was observed in the liver homogenates obtained from this animal group (Fig 4B). When compared to sham-animals, no significant increase in iNOS expression or serum NOx was noticed in HS_50_-rats. In contrast, kidney and liver of HS_50_-rats pretreated with LPS exhibited a pronounced increase of iNOS expression (Fig 4A and 4B) and serum NOx (Fig 4C). Although the pretreatment of sham-operated rats with LPS augmented serum NOx when compared to sham treated with vehicle (Fig 4C), the iNOS expression among these groups was not significantly different (Fig 4A and 4B). In spite of the increased serum levels of NOx in LPS preconditioned animals (sham and HS_50_), the basal MAP was no different among the groups studied (Fig 4D).

### Effect of LPS preconditioning on NF-ĸB pathway

**Fig 5:**
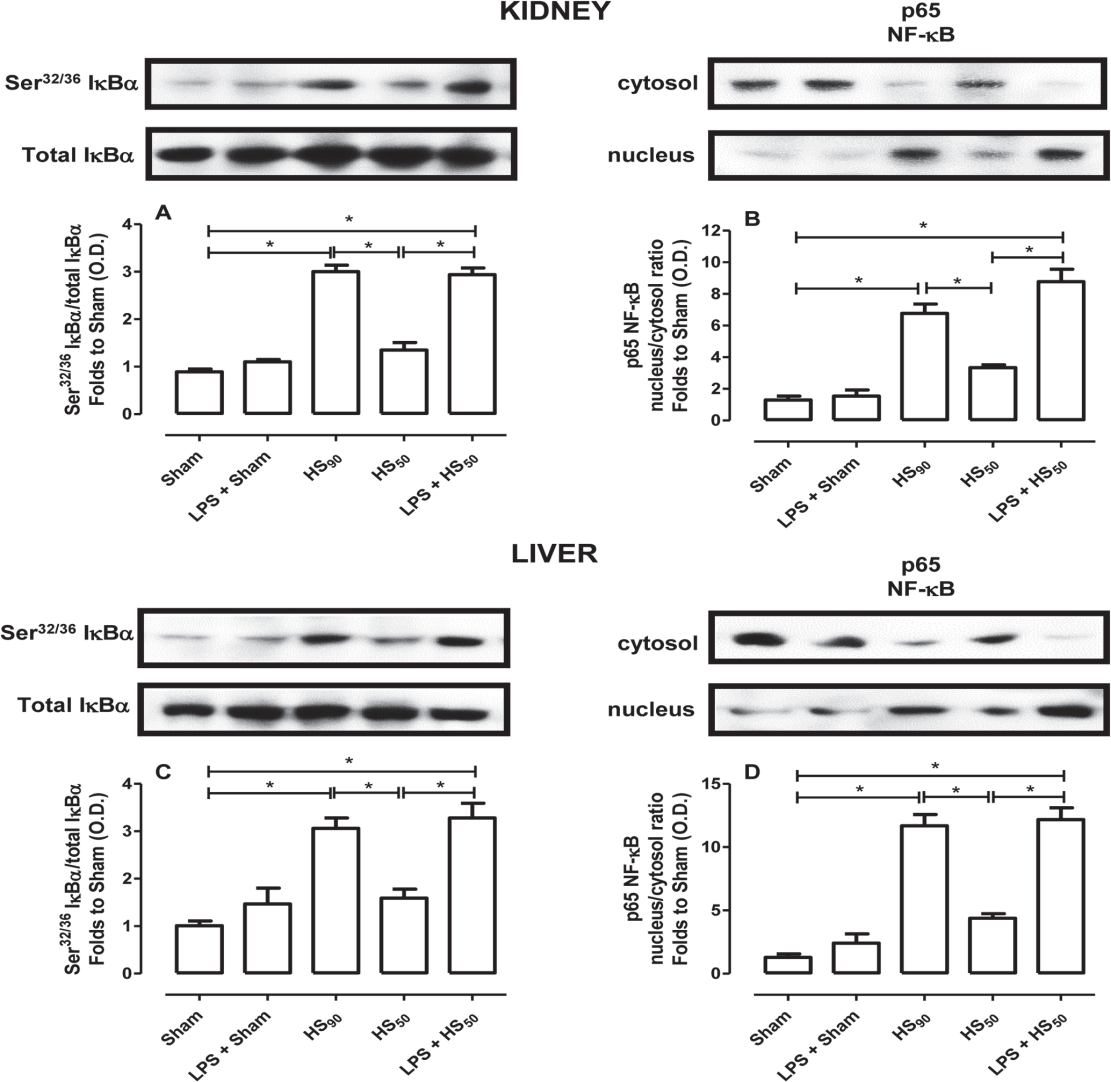
Effect of LPS preconditioning on NF-ĸB activation. Rats were injected vehicle (PBS) or LPS (1 mg/kg) i.p. 24 h prior to HS_90_ or HS_50_. The phosphorylation of IĸBα on Ser^32/36^ (A and C) and the nuclear translocation of the p65 NF-ĸB subunit (B and D) on the kidney (A and B) and liver (C and D) were determined by western blotting. Protein expression was measured as relative optical density (O.D.), corrected for the corresponding β-actin contents and normalized using the related Sham band. Data are expressed as mean ± SEM of n observations. Statistical analysis was performed using one-way ANOVA followed by Bonferroni’s post hoc test. *p < 0.05 among the groups.

As iNOS is a NF-κB-dependent gene, we have also investigated the degree of activation of NF-κB in liver and kidney samples from all animal groups. When compared to sham-operated rats, kidney (Fig 5A and 5B) and liver (Fig 5C and 5D-) homogenates obtained from HS_90_-rats showed a significant increase in the phosphorylation of IκBα on Ser^32/36^ (Fig 5A–5C) and the nuclear translocation of the NF-κB subunit p65 (Fig 5B–5D). In contrast, no difference was noticed between Sham and HS_50_ groups. When compared to HS_50_-rats, LPS preconditioning increased the phosphorylation of IκBα and the activation of NF-κB (Fig 5), exhibiting similar magnitude of increase than HS_90_ group.

### Effect of LPS preconditioning on cytokines production

**Fig 6:**
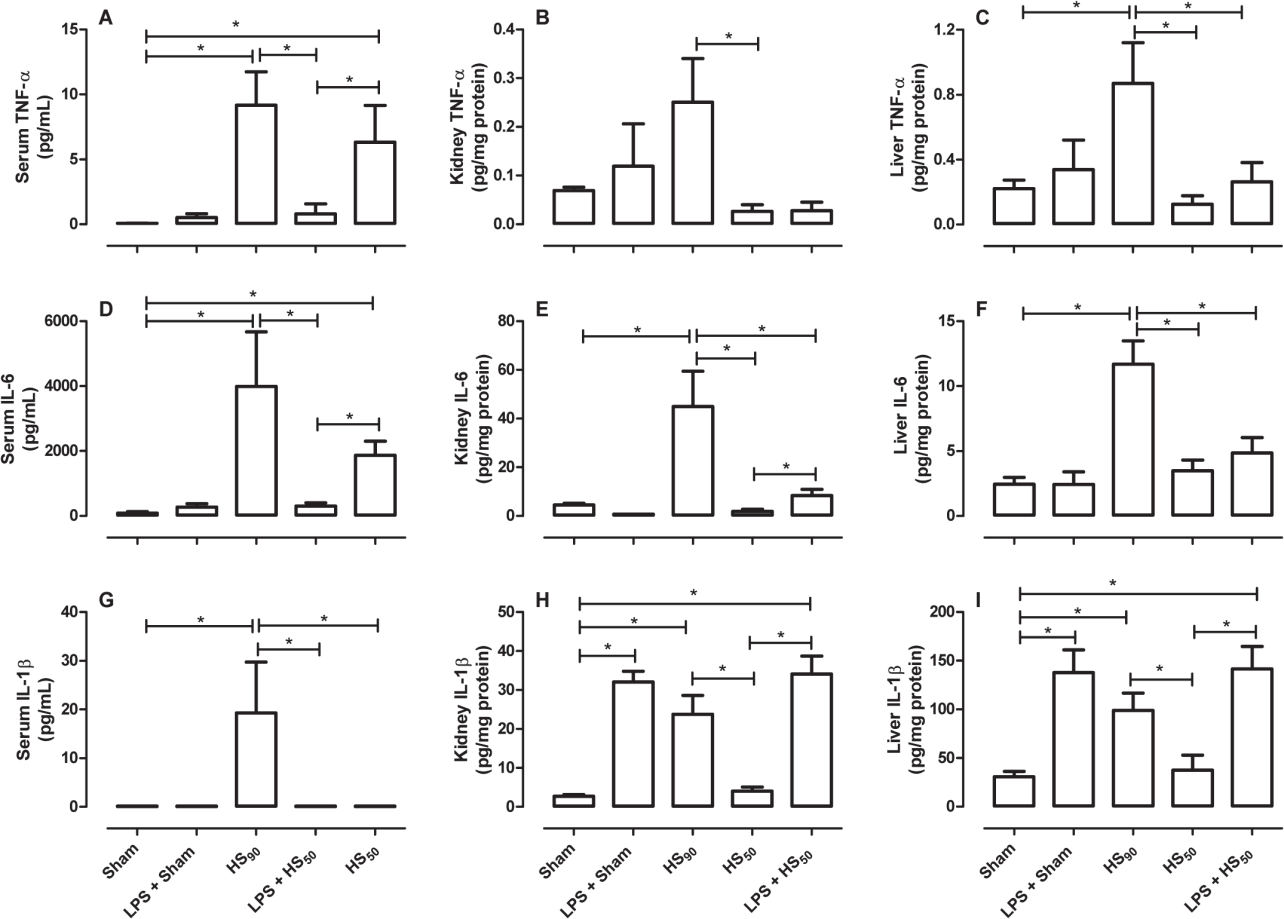
Effect of LPS preconditioning on cytokines production. Rats were injected vehicle (PBS) or LPS (1 mg/kg) i.p. 24 h prior to HS_90_ or HS_50_. Sham animals were used as control. Four hours after resuscitation, serum (A, D, G), kidney (B, E, H) and liver homogenates (C, F, I) were obtained for TNF-α (A-C), IL-6 (D-F) and IL-1β (G-I) determination by ELISA. Data are expressed as mean ± SEM of 6–8 animals. Statistical analysis was performed using one-way ANOVA followed by Bonferroni’s post hoc test. * p < 0.05 among the groups.

When compared with sham-operated animals, HS_90_ resulted in increased concentrations of TNF-α, IL-6 and IL-1β in serum as well as in homogenates prepared from liver and kidney (Fig 6). In contrast, when compared to sham animals, HS_50_ caused no change on any of the cytokines evaluated (Fig 6). Interestingly, compared with HS_50_ group, HS_50_-rats subjected to LPS preconditioning had elevated concentrations of i) TNF-α in serum (Fig 6A), ii) IL-6 in serum and kidney (Fig 6D and 6E), and iii) IL-1β in kidney and liver (Fig 6H and 6I), indicating an aggravated inflammatory response in these animals. When compared to the sham-vehicle group, LPS preconditioning of sham animals only caused a significant increase in the concentrations of IL-1β in kidney and liver (Fig 6H and 6I).

## Discussion

As LPS preconditioning has intriguing and interesting effects in several ischemic-reperfusion-type injury, the present study examined the effect of LPS preconditioning on organ injury/dysfunction induced by HS. Surprisingly, our study demonstrated that preconditioning of rats with a single dose of LPS (1 mg/kg 24 h prior to HS) aggravated the organ injury and dysfunction associated with HS. Preconditioning with LPS caused an excessive activation of NF-κB, induction of iNOS expression and NO production, which in turn, may have contributed to the incapability of preconditioned animals to sustain blood pressure at a level of 30 mmHg during bleeding, reducing the resistance of the animals to HS. Moreover, LPS preconditioned HS-rats exhibited higher concentrations of pro-inflammatory cytokines, which may have a role for the poor outcome following resuscitation.

The hemodynamic decompensation in HS, which occurs after prolonged periods of hemorrhage, is defined as a progressive vasodilation and a continuous decrease in peripheral vascular resistance that ultimately results in death [4, 18]. Interestingly, 90% of the LPS preconditioned animals had to be resuscitated when 25% of the blood was re-injected, which means that rats which had been preconditioned with LPS were more susceptible to develop vascular decompensation during hemorrhage. The decision to resuscitate animals in which 25% of the shed blood had been re-injected to sustain a MAP of 30 mmHg was based on our experience that animals which are not resuscitated at this time point do not survive the entire protocol (data not shown). Therefore, LPS preconditioned rats were maintained at a MAP of 30 mmHg for approximately 50 min, while HS-rats pretreated with vehicle were maintained at an MAP of 30 mmHg for approximately 90 min. Moreover, the amount of blood withdrawn during hemorrhage was also less in HS-rats preconditioned with LPS, which means that the animals reached a low blood pressure faster than both control groups, although the basal MAP was similar in all groups of animals. Thus, our finding (Fig 1) that the organ injury/dysfunction is attenuated in HS-rats that had been preconditioned with LPS is not surprising, as these animals had been subjected to a less severe insult.

Having discovered that the reduction in organ injury/dysfunction in HS-rats preconditioned with LPS was due to the fact that these animals had been subjected to a less severe hemorrhage protocol, we designed a study to account for this effect. Therefore, in order to have a proper control group, we performed a further group in which all animals were resuscitated 50 min after hemorrhage (named HS_50_ group). When both vehicle and LPS-pretreated animals were subjected to an identical period of hemorrhage (50 min), LPS preconditioning worsened the organ injury and dysfunction induced by HS, as well as aggravated the circulatory failure in these animals.

What, then, is the reason for a more pronounced vascular decompensation in rats preconditioned with LPS? Vascular decompensation is associated with induction of iNOS and increasing levels of NO [4, 18]. LPS activates TLR4 and regulates iNOS gene expression and nitric oxide production via the activation of NF-κB [19–21]. Enhanced expression of iNOS has been reported in a) LPS preconditioning, b) in animals that receive LPS-based vaccines, and c) in animals subjected to HS [22–24]. As NO is pivotal for the preservation of microvascular perfusion [5], we have investigated the induction of iNOS in kidney and liver, which are the organs where we had observed a significant injury/dysfunction associated with HS. We report here that, in the absence of preconditioning with LPS, hemorrhagic shock (HS_90_-rats) led to the phosphorylation of Ser^32/36^ on IκBα (indicating IKK activation), IĸBα degradation and consequently translocation of NF-κB p65 to the nucleus (in liver and kidney). This activation of NF-κB led to an increase in iNOS expression. When we employed a shorter hemorrhage protocol (HS_50_) and pretreated these animals with vehicle, we were unable to detect a significant activation of NF-κB and the associated expression of iNOS. In contrast, HS_50_-rats preconditioned with LPS exhibited a dramatic increase in NF-κB activation (also accompanied by the phosphorylation of Ser^32/36^ on IκBα) and iNOS induction in kidney and liver. Indeed, HS_50_-rats preconditioned with LPS had 15-fold and 20-fold higher level of NOx when compared with HS_90_ and HS_50_-rats treated with vehicle, respectively. Most notably, the increase in serum NOx at baseline caused by preconditioning with LPS is more pronounced than the increase in NOx caused by HS itself, which amounts to a 3-fold increase in NOx in either animal group. Thus, we speculate that the dramatic increase in iNOS expression and the resulting excessive NO (NOx) production caused by LPS preconditioning may well account for the fact that these animals decompensate quicker when challenged with HS. Indeed, while total inhibition of NO formation may impair microvascular perfusion and aggravate organ injury [5], inhibition of an enhanced formation of NO prevents, reverts or at least minimizes the delayed vascular decompensation and the hyporeactivity to vasoconstrictor agents during HS [4, 25].

We have not investigated the effects of an iNOS inhibitor in LPS preconditioned rats subjected to HS_50_, because it is already known that the administration of iNOS inhibitors before LPS preconditioning attenuates the effect of preconditioning [19, 22, 26]. In addition, the administration of selective inhibitors of iNOS reduces the vascular decompensation and the organ injury/dysfunction associated with HS [27–29]. Taken together, these findings demonstrated that a) an enhanced formation of NO from iNOS contributes to both vascular decompensation and organ injury/dysfunction in HS, and b) LPS-preconditioning causes a further dramatic increase in iNOS expression and NO formation (secondary to activation of NF-κB), which importantly contributes to the aggravation of both circulatory failure (e.g. vascular decompensation) and organ injury and dysfunction caused by HS.

HS increases TLR4 expression, which activates NF-κB resulting in enhanced formation of pro-inflammatory cytokines. Indeed, LPS preconditioning in animal models of HS and ischemia-reperfusion injury is associated with down-regulation of TLR4, at least at the intestinal mucosa [30–32]. There is good evidence that an enhanced formation of TNF-α drives the excessive formation of pro-inflammatory cytokines resulting in organ injury and death in animals and man with septic or hemorrhagic shock [30, 31]. We report here that hemorrhagic shock (HS_90_ group) results in the excessive formation of TNF-α, IL-6 and IL-1β (in serum, kidney and liver homogenates), and elevation of all three of these cytokines contributes to the development of MOF and death in HS [33–36].

We found no increase in TNF-α in a) sham rats, b) sham-rats preconditioned with LPS or c) rats subjected to the shorter hemorrhage protocol (HS_50_). In contrast, HS_50_ rats preconditioned with LPS developed a dramatic increase in serum TNF-α, and the magnitude of the observed rise in TNF-α in these animals was similar to that found in the HS_90_ group. Indeed the rise in serum TNF-α caused by HS_50_ in rats pre-conditioned with LPS was 7–8 folds higher than in HS_50_ rats pre-treated with vehicle. We observed no significant change in TNF-α in kidney and liver homogenates from a) sham rats, b) sham-rats preconditioned with LPS, c) rats subjected to the shorter hemorrhage protocol (HS_50_) treated with vehicle, or d) HS_50_-rats preconditioned with LPS. However, the lack of ‘local’ production of TNF-α does not mean that TNF-α does not, in principle, contribute to the development of organ dysfunction in HS, as it is known that patients who either produce excessive amounts of cytokines (resulting in raised serum TNF-α levels) or cannot clear them from the circulation have an increased risk of MOF and death [30].

When compared to HS_50_-rats treated with vehicle, HS_50_-rats preconditioned with LPS showed increased levels of IL-6 (serum and kidney). Most notably, elevated serum IL-6 concentrations predict the development of organ failure in trauma-hemorrhage patients [32]. Thus, preconditioning with LPS enhanced serum IL-6, which together with the enhanced levels of TNF-α in these animals, may have importantly contributed to the excessive organ dysfunction observed in HS_50_-rats preconditioned with LPS.

An increase in serum IL-1β contributes for the development of SIRS and organ dysfunction following hemorrhage [34–36]. We found no increase in IL-1β in homogenates of both kidney or liver of rats subjected to the shorter hemorrhage protocol (HS_50_-rats) pre-treated with vehicle. In contrast, HS_50_-rats that had been preconditioned with LPS exhibited marked increases of IL-1β in homogenates of both kidney and liver. Thus, preconditioning with LPS enhanced serum IL-6 and serum TNF-α as well as tissue (kidney/liver) IL-1β, all of which may have importantly contributed to the excessive organ dysfunction observed in HS_50_-rats preconditioned with LPS.

Chang and colleagues have recently shown that LPS preconditioning ameliorates intestinal injury in HS [32]. They showed that LPS preconditioning reduced TLR4 expression in the intestine resulting in a reduced inflammatory response. However, the animals were submitted to MAP of 35–40 mmHg, which is higher than our values of blood pressure (30 ± 2 mmHg), and the authors did not mention the volume of shed blood in the different experimental groups. Moreover, tissue-specific differences exist in LPS preconditioning [23]. Therefore, the success of the study of Chang and colleagues might be organ specific and may be due to a repeated dose regimen of LPS used (0.1 mg/kg i.p. per day for 5 consecutive days), which differs from the LPS-preconditioning protocol employed here (1 mg/kg i.p. at 24 h before HS). Although different LPS-preconditioning protocols (e.g. repetitive vs single administration) employing different doses of LPS do result in variable degrees of tolerance (especially when LPS is used as the ‘second-hit) [37], we opted for a single dose of LPS given 24 h before HS, as this experimental protocol is effective in reducing the injury caused by a subsequent ischemic insult [9; 11; 15]. The LPS preconditioning was not effective in our model of HS due to the early cardiovascular decompensation, which is not necessarily related to the mechanisms underlying organ protection or immune tolerance. Thus, the main importance of our study is to provide some insight into the reasons why preconditioning with LPS in HS may not always lead to beneficial results and, indeed, as shown by us may result in an adverse outcome.

In conclusion, we demonstrated that LPS preconditioning led to a rapid and more pronounced vascular decompensation in HS, which well may be secondary to an excessive formation of NO associated with an increased expression of iNOS in these animals. When the length of hemorrhage was controlled to account for this effect, LPS preconditioning enhanced the organ injury/dysfunction and the circulatory failure caused by HS. This effect may be, at least in part, due to the exacerbated activation (by LPS-preconditioning) of NF-κB leading to increased formation of pro-inflammatory cytokines such as TNF-α, IL-1β and IL-6. Specifically, preconditioning with LPS enhanced serum IL-6 and serum TNF-α as well as tissue (kidney/liver) IL-1β, all of which may have importantly contributed to the excessive organ dysfunction observed in HS_50_-rats preconditioned with LPS.
